# Polymorphism of Sooty-fronted Spinetail (*Synallaxis frontalis* Aves: Furnariidae): Evidence of chromosomal rearrangements by pericentric inversion in autosomal macrochromosomes

**DOI:** 10.1590/1678-4685-GMB-2018-0039

**Published:** 2019-03-11

**Authors:** Marcelo Santos de Souza, Suziane Alves Barcellos, Alice Lemos Costa, Rafael Kretschmer, Analía Del Valle Garnero, Ricardo José Gunski

**Affiliations:** 1 Universidade Federal do Pampa Universidade Federal do Pampa Programa de Pós-graduação em Ciências Biológicas São GabrielRS Brazil Programa de Pós-graduação em Ciências Biológicas, PPGCB, Universidade Federal do Pampa, São Gabriel, RS, Brazil; 2 Universidade Federal do Pampa Universidade Federal do Pampa Laboratório de Diversidade Genética Animal São GabrielRS Brazil Laboratório de Diversidade Genética Animal, Universidade Federal do Pampa, São Gabriel, RS, Brazil; 3 Universidade Federal do Rio Grande do Sul Universidade Federal do Rio Grande do Sul Programa de Pós-Graduação em Genética e Biologia Molecular Porto AlegreRS Brazil Programa de Pós-Graduação em Genética e Biologia Molecular, PPGBM, Universidade Federal do Rio Grande do Sul, UFRGS, Porto Alegre, RS, Brazil

**Keywords:** Chromosomal polymorphism, evolution, cytogenetics

## Abstract

The Passeriformes is the most diverse and cytogenetically well-known clade of birds, comprising approximately 5,000 species. The sooty-fronted spinetail (*Synallaxis frontalis* Aves: Furnariidae) species, which belongs to the order Passeriformes, is typically found in South America, where it is widely distributed. Polymorphisms provide genetic variability, important for several evolutionary processes, including speciation and adaptation to the environment. The aim of this work was to analyze the possible cytotypes and systemic events involved in the species polymorphism. Of the sampled 19 individuals, two thirds were polymorphic, an event supposedly linked to mutations resulting from genomic evolution that can be transmitted hereditarily. A chromosomal polymorphism was detected between the 1st and 3rdpairs of autosomal macrochromosomes. This type of polymorphism is related to a pericentric inversion in regions involving chromosomal rearrangements. Differently from other polymorphism studies that report a link between polymorphic chromosomes and phenotypic changes, *S. frontalis* did not present any morphological variation in the sampled individuals.

## Introduction

The order Passeriformes comprises approximately 5,000 species and is the most diverse order within the class Aves. Few studies have demonstrated morphological variations, called polymorphism, in karyotypes of the same species. Polymorphism can be considered a gene marking mechanism, since they are hereditarily transmitted from the progenitor to the offspring, when viable (Balasubramanian *et al.*, 2004). Among all Passeriformes karyotypes described so far, only five species presented chromosomal polymorphism (Thorneycroft, 1966; Shields 1973, 1976; Thomas *et al.*, 2008; Itoh *et al.*, 2011; Kretschmer *et al.*, 2018)

Breakpoints are regions that accumulate hot spots that are being used and reused during genomic evolution (Glover and Stein, 1988). The fragile sites are evolutionary breakpoints, highly conserved and transmitted from a common ancestor (Ruiz-Herrera and Robinson, 2007; Brown and O’Neill, 2010).

Olsson *et al.* (2001) believe that polymorphisms are responsible, in part, for genetic diversity, once they result in different phenotypes. The first chromosomal polymorphism described in birds was reported in *Zonotrichia albicollis* (Gmelin, 1789)*,* and it is likely due to pericentric inversions in the second and third pairs of chromosomes (Thorneycroft, 1966). In recent studies with this species, Thomas *et al.* (2008) discuss the relationship of this polymorphism with divergences in plumage and social behavior.

Shields (1973, 1976) observed in *Junco hyemalis* (Linnaeus, 1758) a pericentric inversion involving the second and fifth chromosome pairs, but this variation was never seen simultaneously in the same karyotype. No significant change was found in the polymorphic individuals (physical or behavioral). The same results were described by Manolache (1974) in *Coturnix coturnix coturnix* (Linnaeus, 1758), who demonstrated morphological differences in the second pair of autosomal chromosomes. Chromosomal translocation involving a chromosome in the third pair in *Oriolus xanthornus* (Horsfield, 1821) was reported as an important polymorphism, but it did not cause changes in the phenotype (Ansari and Kaul, 1978).

Genetic variability is fundamental for basic processes of natural selection; it occurs at individual levels and may or may not improve individual fitness. This mechanism occurs due to chromosomal changes, derived from translocations, inversions, mutations, deletions, and duplications in specific regions of the genome (Kageyama and Jacob, 1980; Nascimento *et al.*, 1990).

The Furnariidae family belongs to the order Passeriformes, and it is currently composed of about 236 described species distributed into 71 genera, most of them endemic to South America and distributed in several ecological niches. Among the Furnariidae family, the genus *Synallaxis* is the most diverse, presenting 33 described species (Derryberry *et al.*, 2011; Sigrist, 2013). Despite this diversity, cytogenetic data are available for only five species: *Furnarius rufus* Gmelin, 1788, *Lochmias nematura* Lichtenstein, 1823 (Lucca and Rocha, 1992), *Sittasomus griseicapillus* Vieillot, 1818, and *Lepidocolaptes angustirostris* Vieillot, 1818 (Barbosa *et al.*, 2013).

All karyotyped Furnariidae species have the same diploid number (2n=82) and show remarkable similarity in the chromosomal morphology, presenting few variations only in machrocromosomes, which are predominantly telocentric and acrocentric. Nevertheless, *Synallaxis frontalis* (Pelzeln, 1859) individuals present a striking characteristic of rearrangements that are responsible for small morphological variations in the chromosomal arms (Kretschmer *et al.*, 2018).

The chromosomal variations observed suggest a high chromosomal fragility, responsible for facilitating the occurrence of intraspecific chromosomal polymorphism. However, chromosomal polymorphism in individuals of a population may alter the karyotype pattern of these specimens (Teodoro-Pardo, 2007).

Due to the chromosomal morphological variability in *Synallaxis frontalis*, this study aimed to analyze the possible karyotypes and systemic mechanisms involved in the polymorphism.

## Materials and Methods

Nineteen specimens of *Synallaxis frontalis* (10 males and 9 females) were collected in the municipality of São Gabriel, RS, under the SISBIO licenses nº 44173-1 and nº 33860-1, and CEUA/UNIPAMPA, under protocol no. 026/2012. Captures occurred from 2013 to 2017, using a mist net. We also analyzed a family composed of a couple and four offspring (2 males and 2 females), called SFR family in this paper. The four individuals were captured in a non-random manner from the same nest.

### Tissue cultures and chromosome preparation

Metaphases were obtained using short term culture of bone marrow (Garnero and Gunski, 2000) and fibroblast from biopsies, according to the Sasaki *et al.* (1968) method with modifications, in which samples were fractionated mechanically and incubated in collagenase solution (0.0186 g in 4 mL of DMEM medium), for 1 h at 37 °C for cell dissociation. After centrifugation and discarding the supernatant, 5 mL of DMEM supplemented with antibiotics and fetal bovine serum (15%) were added, and the material was transferred to cell culture flasks. Both methods included colcemid incubation, hypotonic treatment, and fixation with methanol:acetic acid (3:1).

### Conventional cytogenetics: Giemsa staining, C-banding, G-banding and karyotyping

The distribution of heterochromatic blocks was analyzed by C-banding (Ledesma *et al.*, 2006), with modifications. After incubation at 60 °C for 1 h, the slides were treated with 0.2 N HCl for 10 min, 50% Ba (OH2) for 15 min at 37 °C, 0.01 N HCl for 2 min, 2 SSC at 60 °C for 1h and 30 min, and stained with Giemsa (5% in 0.07M phosphate buffer, pH 6.8) for 15 min. Karyotypes were arranged according to Levan *et al.* (1964), and the chromosomes were classified as metacentric, submetacentric, subtelocentric, and telocentric according to arm ratio (r) and centromeric index (i). The G-band analysis and patterns were done according to Howe *et al.* (2014).

### Microscopic analysis

We analyzed approximately 30 metaphase spreads per individual in an optical microscope (OLYMPUS DP53) to confirm chromosome number and morphology. For composing karyotype figures, Corel Draw^®^ 12 was used.

## Results

**Figure 1:**
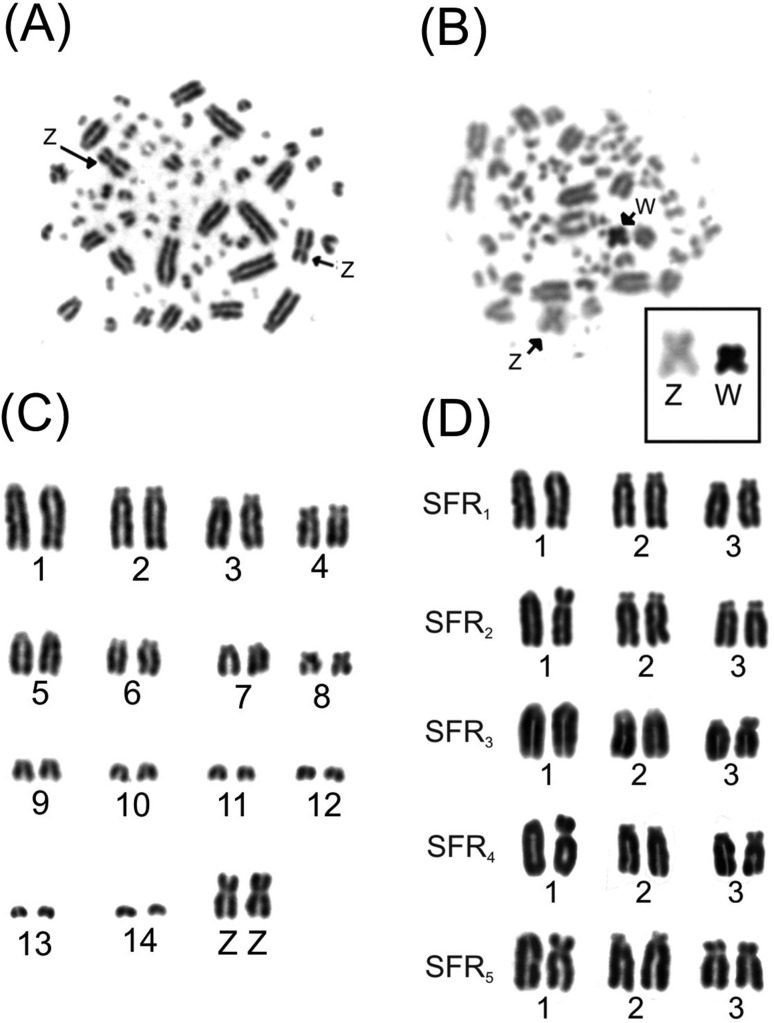
Classical cytogenetics techniques in *Synallaxis frontalis* (SFR). (A) Conventional staining of metaphases from a male; arrows indicate the sexual chromosome Z. (B) Partial karyotype containing macrochromosomes, microchromosomes, and sex chromosomes. (C). C-banding of a female showing the W sex chromosome, entirely heterochromatic. (D) Five distinct cytotypes found in samples.

*Synallaxis frontalis* presented the diploid number of 82 chromosomes, with 11 pairs of macrochromosomes, including sex chromosomes Z and W, and 30 pairs of microchromosomes (Figure 1A-C). Of all the cytotypes analyzed in this study, the second, fifth, sixth and seventh pairs presented a subtelocentric morphology. Differently, the fourth pair was submetacentric, the eighth pair and the sex chromosomes were metacentric, and the remaining others were telocentric.

We found a chromosomal polymorphism originated from a break followed by a pericentric inversion in the first and third autosomal pairs. Five different cytotypes were observed, these being the standard telocrocentric in the 1^st^ pair and a subtelocentric in the 3^rd^ (Table 1). All the cytotypes found in *S. frontalis* are described in Figure 1D.

**Table 1:** Cytotype frequency in *Synallaxis frontalis*.

	1st and 3rd chromosome pairs	Male	Female	%
A	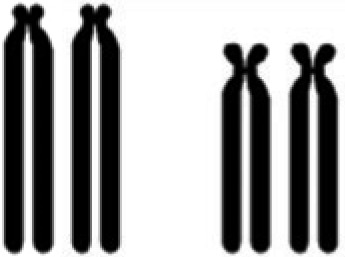	6	1	36.84
B	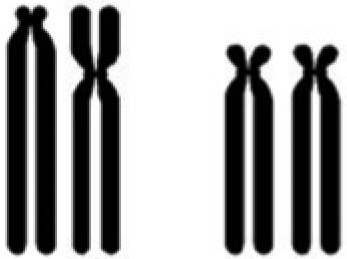	2	1	15.79
C	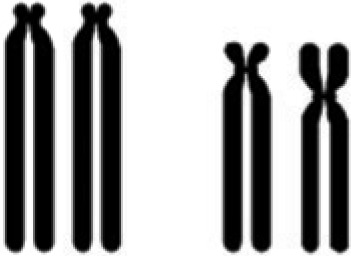	2	5	36.84
D	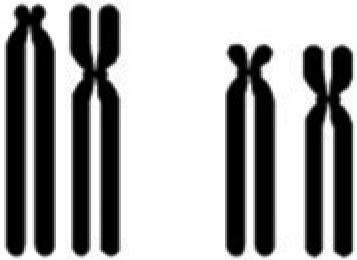	-	1	5.26
E	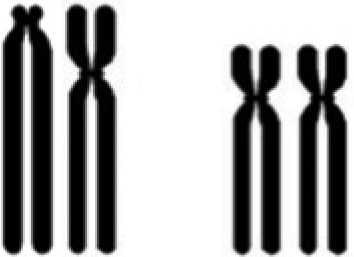	-	1	5.26
	Total	10	9	100

A= Standard; B= Polymorphic heterozygous for the 1st pair; C= Polymorphic heterozygous for the 3rd pair; Polymorphic heterozygous for 1st and 3rd pairs; E= Polymorphic heterozygous for the 1st pair and polymorphic homozygous for the 3rd pair.

The C-banding analyses showed blocks of constitutive heterochromatin in the centromeric regions of some of the macrochromosomes and microchromosomes. The sex chromosome W was entirely heterochromatic (Figure 1C).

**Figure 2:**
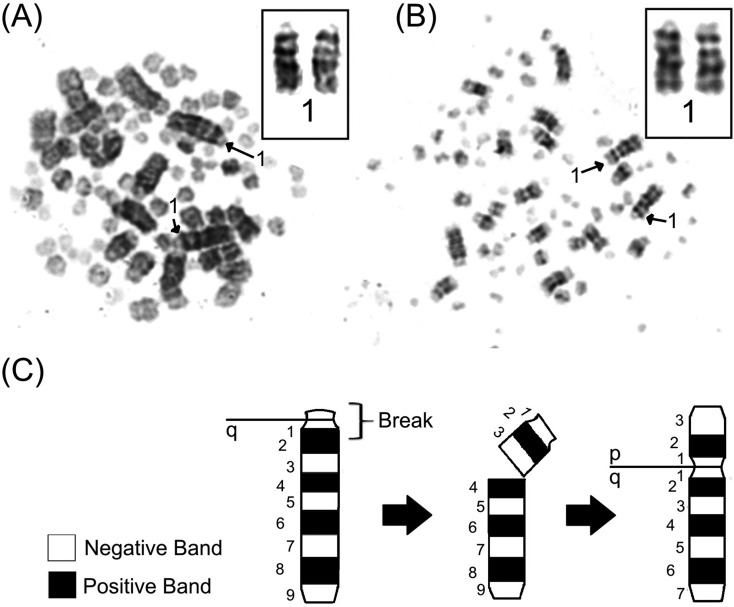
G-banding patterns found in standard and polymorphic individuals. Metaphasis of a standard (A) and polymorphic (B) bird; arrows indicate the first chromosome pair expanded in the box. (C) Schematic illustration of pericentric inversion.

The G-banding pattern in *S. frontalis* had evidence of pericentromeric inversions in the heteromorphic chromosomes. The inversions involved one positive band, two negative bands, and the centromere in the first and third pairs (Figure 2).

Among the 19 sampled individuals, two thirds presented polymorphisms in the first, third, or in both pairs; generally, the frequency of polymorphic individuals was higher than that of non-polymorphic ones in both sexes. Heterozygous individuals were more abundant in polymorphism than polymorphic homozygotes or standard ones.

**Figure 3:**
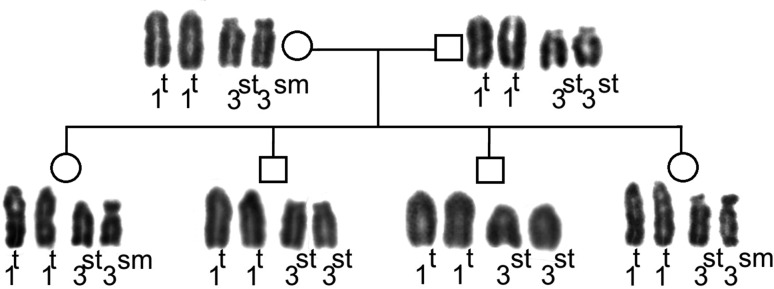
Heredogram of the SFR family. Circles represent the females and squares, the males. The numbers refer to chromosome pairs. t: telocentric; st: subtelocentric; sm: submetacentric.

Two cytotypes were found in the SFR family. Females presented a heterozygous polymorphism in the third pair, while the males presented a standard cytotype (Figure 3).

## Discussion

The diploid and chromosomal morphologies found in the sampled birds were similar to those of Furnariidae species (chromosome number around 80), as described in the literature (Barbosa *et al.*, 2013). It is known that the first and third chromosome pairs are heteromorphic in this species, and we found in some individuals short arms of different lengths in pairs 1 and 3 (Figure 1) (Kretschmer *et al.*, 2018).

One in three analyzed birds presented the standard karyotype, and the other two had karyotypes varying within four different cytotypes. The frequency of polymorphism was very high. However, a reasonable portion of individuals had a standard cytotype. This cytotype was thus defined based on cytogenetic and phylogenetic data. The phylogeny of the Furnariidae family defines the genus *Synallaxis* as slightly derived within the group (Irestedt *et al.*, 2009).

In a recent work, the morphological variations in chromosome pairs 1 and 3 were defined as heteromorphism, since it would be necessary to analyze a larger sample to define them as polymorphism (Kretschmer *et al.*, 2018). Nevertheless, our study showed that variations in morphology are polymorphisms by the distribution and fixation of different cytotypes in the specimens sampled.

From the 19 analyzed birds, 12 presented some of the described polymorphic cytotypes (Table 1). The most common polymorphic cytotype (36% approximately) was the heterozygous polymorphism in the third chromosomal pair. Less common cytotypes observed were heterozygous polymorphisms for both chromosome pairs and homozygous polymorphisms for any of the pairs. This data together with the karyotype analysis of the species emphasizes the possibility that there are no pre- or post-zygotic barriers to any of the possible polymorphic combinations in *S. frontalis* (Figure 1).

According to Kretschmer *et al.* (2018)*,* hybridization with *Leucopternis albicollis* (LAL) probes has revealed the possible mechanism responsible for its specific morphological variation, assuming that both polymorphisms would have arisen either by pericentric inversions or centromere repositioning. Indeed, our study has shown through G-banding that the origin of the polymorphic chromosomal pairs comes from pericentric inversions, where the bands 1, 2 and 3 of the q-arm of a single chromosome in pair 1 became bands 1, 2 and 3 of the p-arm (Figure 2C). This inversion includes the centromere, discarding the possibility of centromere repositioning. The same also occurred in the third chromosomal pair of this species.

Genetic changes related to the *S*. *frontalis* polymorphism could be favorable for this species, as indicated by the high frequency of polymorphic individuals found in this study. However, further studies are needed to confirm this. Such mechanisms are associated with evolutionary phases and events of speciation, as reported in Frankham *et al.* (2002).

A polymorphism is inherited when there is no pre- or post-zygotic barrier (Balasubramanian *et al.*, 2004). Based on the SFR family analysis, we can infer that there is no pre-zygotic barrier, because the progenitors presented the polymorphic, as well as the standard cytotypes. Furthermore, the offspring showed two chromosomal morphotypes, confirming that the polymorphism is inheritable. Also, we highlight that the polymorphism mechanism in *S*. *frontalis* is not related to sex.

However, it is difficult to determine the size or the number of populations of this species because it is widely distributed in South America, and it is unlikely that there are possible genetic or geographical barriers separating populations.

It is assumed that the presence of breakpoints or hot spots in the chromosomal regions of this species are inversions. This could be considered an advantageous evolutionary characteristic for *S. frontalis*; however, it is not possible to estimate or argue that speciation occurred in the specimens analyzed. During genomic evolution of a species, the accumulation of breakpoints is frequent (Glover and Stein, 1988). The inversions found in the polymorphic individuals of this study demonstrate how important this system is for the genetic variability and maintenance of mechanisms that originate the polymorphism within the population.

In *S. frontalis* there is no apparent phenotypic characteristic that differentiates individuals as polymorphisms (Thorneycroft, 1966; Thomas *et al.*, 2008). In contrast, *Zonotrichia albicollis* has a polymorphism that causes changes in behavior and plumage. Thus, as also seen in the SFR family, one can conclude that there is no visible sexual or environmental selection of polymorphic or non-polymorphic individuals living in sympatry.

In conclusion, the present work indicates that there is striking diversity in *S. frontallis* karyotype composition, leading to several cytotypes. This variability may be the result of evolutionary processes. The study of such species can contribute to the understanding of how phylogenetic diversity occurs, and thus the preservation and further study of this species is important.
